# Neurological manifestations of post-acute sequelae of COVID-19: which liquid biomarker should we use?

**DOI:** 10.3389/fneur.2023.1233192

**Published:** 2023-07-21

**Authors:** Dominique Comeau, Mykella Martin, Gilles A. Robichaud, Ludivine Chamard-Witkowski

**Affiliations:** ^1^Dr. Georges-L. Dumont University Hospital Centre, Clinical Research Sector, Vitalité Health Network, Moncton, NB, Canada; ^2^Centre de Formation médicale du Nouveau-Brunswick, Université de Sherbrooke, Moncton, NB, Canada; ^3^Department of Chemistry and Biochemistry, Université de Moncton, Moncton, NB, Canada; ^4^The New Brunswick Center for Precision Medicine, Moncton, NB, Canada; ^5^The Atlantic Cancer Research Institute, Moncton, NB, Canada; ^6^Department of Neurology, Dr. Georges-L. Dumont University Hospital Centre, Moncton, NB, Canada

**Keywords:** neuro-PASC, biomarkers, NfL, GFAP, IL-6, IL-10, TNF-α, CPR

## Abstract

Long COVID syndrome, also known as post-acute sequelae of COVID-19 (PASC), is characterized by persistent symptoms lasting 3–12 weeks post SARS-CoV-2 infection. Patients suffering from PASC can display a myriad of symptoms that greatly diminish quality of life, the most frequent being neuropsychiatric. Thus, there is an eminent need to diagnose and treat PASC related neuropsychiatric manifestation (neuro-PASC). Evidence suggests that liquid biomarkers could potentially be used in the diagnosis and monitoring of patients. Undoubtedly, such biomarkers would greatly benefit clinicians in the management of patients; however, it remains unclear if these can be reliably used in this context. In this mini review, we highlight promising liquid (blood and cerebrospinal fluid) biomarkers, namely, neuronal injury biomarkers NfL, GFAP, and tau proteins as well as neuroinflammatory biomarkers IL-6, IL-10, TNF-α, and CPR associated with neuro-PASC and discuss their limitations in clinical applicability.

## 1. Introduction

Persistent neurological and psychiatric symptoms associated with coronavirus disease 2019 (COVID-19), referred to as neurological symptoms of Post-Acute Sequelae of COVID-19 (neuro-PASC), has garnered much attention since the beginning of the pandemic (1–4). Symptoms persisting 3–12 weeks after infection with severe acute respiratory syndrome coronavirus 2 (SARS-CoV-2) include fatigue, cognitive dysfunction, sleep disorders, anxiety disorders and dementia, among others (1, 4–7). Neurological symptoms represent some of the most debilitating symptoms of PASC (1). Furthermore, the commonality of these symptoms signals an urgent need for clinically relevant tools for the diagnosis and management of the illness (1, 5, 6, 8, 9). Opportune and accurate diagnosis of neurological disease in clinical practice is of great importance; in this context, biomarkers may represent a potentially viable diagnostic tool. Biomarkers could be used in guiding clinical diagnosis, prognosis, evaluating disease stage and monitoring disease progression or disease-modifying therapies. Furthermore, identifying reliable biomarkers in neuro-PASC could avoid misdiagnosis which can lead to suboptimal care and avoid unnecessary care-seeking and costly investigations due to diagnostic uncertainty (7). Liquid biomarkers have proven to be extremely useful in the assessment of neurological disease (10) and as indicators of general neurodegeneration and glial activation (11). More specifically, liquid biomarkers from the blood or cerebrospinal fluid (CSF) are particularly practical as they are cost-affective, highly specific and sampling is minimally invasive (12). The aim of this review is to summarize the current knowledge about clinically relevant biomarkers in neuro-PASC and their potential applicability and limitations. We focused our mini-review on the biomarkers that had been the most described and reported in the literature. These biomarkers include neuronal injury biomarkers neurofilament light chain (NfL), glial fibrillary acidic protein (GFAP) and tau proteins as well as inflammatory markers Interleukin (IL)-6, IL-10, tumor necrosis factor alpha (TNF-α) and C-Reactive Protein (CRP).

## 2. Potential neuro-PASC biomarkers

### 2.1. Neuronal injury biomarkers

#### 2.1.1. NfL and GFAP

Plasma NfL and GFAP are well established biomarkers of central nervous system disease diagnosis and progression (13, 14). NfL is a major structural protein only expressed in neurons and an indicator of axonal degeneration and injury used as a blood and CSF biomarker in the assessment of neurodegenerative diseases including frontotemporal lobal degeneration, amyotrophic lateral sclerosis, Alzheimer’s disease (AD), Multiple Sclerosis and primary tauopathies (15–17). Levels of NfL are associated with the intensity of on-going neurodegeneration (17–19) as well as the clinical effectiveness of treatment modalities (20, 21), making it an invaluable clinical tool. GFAP is also an important blood and CSF biomarker. GFAP is an astrocytic intermediate filament which signals astrocytic damage or activation, the presence of which is found in neurodegenerative diseases (22–25) and neuroinflammatory conditions (26, 27).

NfL and GFAP have been found to be elevated in the blood and CSF of patients with COVID-19 as well as in patients with COVID-19 related neurological symptoms (neuro-COVID-19) (28–43). An association between these biomarkers and COVID-19 has been demonstrated during the acute phase of the disease; levels are notably increased in severe cases with neurological involvement and unfavorable outcome (30, 35, 39, 44, 45). Demonstrably, NfL and GFAP were found to be elevated in deceased hospitalized COVID-19 patients (32, 36) and were higher in this cohort when compared to convalescent patients (32). A longitudinal study measuring the trajectories of GFAP and NfL found that patients with severe disease presented an early peak of GFAP during the acute phase which quickly resolved within the first 21 days, and NfL levels were maintained past the 3-week mark (39). Unfortunately, given the severity of the illness, a full neurological and cognitive evaluation was not feasible in this cohort, nor was long-term follow up to evaluate the presence of neuro-PASC in these individuals. In patients with self-reported neuro-PASC (mostly trouble concentrating, headache and dizziness) approximately 4 months after initial infection, plasma NfL and GFAP were measured at early (< 90 days) and late (> 90 days) recovery and compared to levels in patients who did not go on to report neuro-PASC (46). At early recovery, those reporting neuro-PASC symptoms had elevated GFAP but no changes in NfL, and during late recovery neither GFAP nor NfL levels were elevated. Furthermore, there were no significant difference between the two groups at either time point when considering the presence of neurological symptoms during acute infection. Taken together, this may support the possibility of early CNS injury without ongoing neurologic injury even though clinical symptoms persist (46). Irrespective of disease severity, levels of NfL and GFAP were also found to steadily decrease over time and normalize around the 6-month mark (40). In a subset of patients, although levels returned to normal, neurological symptoms persisted, namely, fatigue, brain-fog, and changes in cognition (memory loss and lack of concentration) (40); furthermore, these persistent symptoms were also not correlated to biomarker concentration during the acute phase of the disease. Evidently, trajectories and timing for these biomarkers remains inconsistent between studies (39–41, 44, 46, 47).

Levels of NfL and GFAP were also found increased in mild-to-moderate COVID-19 without evidence of neurological symptoms (29, 44). And, although associated with disease severity, an increase in GFAP in COVID-19 patients was also not associated to neurological symptoms (38). Similarly, NfL was also elevated in the serum of patients without overt neurological manifestations (35, 42). Indeed, in another study, patients with elevated NfL and GFAP did not report persistent neurological disorders (32). In a long-term follow up study (6 months), decreased levels of serum NfL also did not correlate with persistent neurological symptoms or lack thereof (48). Plasma NfL and GFAP was also assessed in hospitalized and non-hospitalized COVID-19 patients with neuro-PASC (41). In this population, both previously hospitalized and non-hospitalized patients experienced decreased quality of life measures (PROMIS) and cognitive dysfunction (NIH Toolbox T scores). Notably, a higher neuroglial score (GFAP/NfL ratio) correlated with increased patient reported anxiety/depression and data suggested that neuro-PASC patients have decreased quality of life irrespective of disease severity. An important caveat to this study was the lack of a control population, namely, patients with COVID-19 but with no neurological symptoms (41). Boni et al. found that in a subgroup of neuro-PASC patients, persistent headaches were not associated to increased NfL and GFAP levels, potentially indicating that this symptom may not be a sign of underlying neuronal damage or neuroinflammation (49). Taken together, the literature is to some extent limited and at variance for the use of these biomarkers in neuro-PASC.

#### 2.1.2. Tau proteins

Tau is a microtubule-associated protein involved in microtubule assembly and stability in CNS axons. Neuronal neurofibrillary tangles and neuropil threads containing hyperphosphorylated tau are pathological features of AD (50). Soluble tau found in CSF, namely, total tau (T-tau) and phosphorylated tau at threonine 181 (p-tau181) have been widely studied in AD (51). Phosphorylated tau has also been reliably detected in blood (52–55). These biomarkers have also been found in neuro-COVID-19 patients (33, 36, 37, 43, 56). COVID-19 patients with new neurological events during hospitalization or presenting with encephalopathy had elevated plasma T-tau and p-tau181 in comparison to patients without these clinical entities. A rise in T-tau and p-tau181 also correlated with symptom severity (36). It was shown that Tau protein levels at admission may also accurately predict fatal outcome (33) although it was not related to ICU transfers (33). A significant correlation between p-tau181, NfL, GFAP levels at admission was also identified; this was however not observed with other inflammatory biomarkers, namely, IL-6, CRP, or ferritin (36). Furthermore, elevated p-tau181 was associated to increased admission, and elevated T-tau was associated with a lower rate of discharge home (36) and in hospital death (36). Conversely, CSF T-tau has been shown to be increased in neuro-COVID-19 patients but not associated to clinical outcomes (45). Paterson et al. found that T-tau and p-tau were also not significantly elevated in the CSF of neuro-COVID-19 patients when compared to non-COVID-19 controls (47). Increased levels of T-tau and p-tau181 have however been correlated with NfL levels (37, 56), notably in patients that report neurological sequelae (56). To date, there are no studies evaluating these biomarkers in neuro-PASC, specifically.

### 2.2. Inflammatory biomarkers

#### 2.2.1. IL-6, IL-10, TNF-α, and CPR

Although the pathophysiologic processes of PASC are not fully understood, immune activation has been proposed to play an important role in the biology of the disease (57, 58); notably, inflammatory biomarkers have been associated with persisting symptoms (57, 59), and major contributing factors in neuropathological processes (60). Namely, IL-6, IL-10, TNF-α and CRP (61, 62) were found to be elevated in the serum of patients with COVID-19 (46, 61, 63–66) and IL-6, IL-10, and CRP have been found to correlate with symptom severity (61, 67). Deceased COVID-19 patients were shown to have higher levels of IL-6 and CRP and were associated to poor clinical outcome and severe organ failure (63). Furthermore, patients with neurological symptoms had increased levels of IL-10 (68) and IL-6 (46). Encephalopathy and inflammatory neurological diseases such as encephalitis, meningitis, acute myelitis was associated with an increase in CSF IL-6 levels (64). It is to be noted that patients only presenting headache as a persistent symptom did not reveal increased inflammatory biomarkers (64). This may suggest that more severe neurological conditions may be correlated with inflammatory process and biomarker expression. TNF-α levels were higher in neuro-PASC patients (46), but when compared to ICU patients, levels did not differ (68) suggesting that ICU patients may had an underlying inflammatory process that could not be discriminated from COVID-19 neurological sequalae. In a study examining neuronal-enriched extracellular vesicles in the plasma of COVID-19 patients 21 days after illness onset, no difference was observed in TNF-α between patients with and without neurological symptoms, which were primarily related to cognitive impairment (56). In contrast, IL-6 tended to be higher (56). In patients with self-reported neuro-PASC, plasma IL-6 and TNF-α measured at late (> 90 days) recovery were significantly higher compared to levels in patients who did not go on to report neuro-PASC symptoms (46). This suggest that inflammation is still present even after infection resolution and may be related to persistent immune response (46). IL-10, TNF-α, CRP and IL-6 have potential diagnostic value for COVID-19 (65); however, evidence supporting their utility in neuro-PASC is presently sparse.

## 3. Limitations

The definition of the timeline for PASC is not unanimous (6). The World Health Organization suggested that post-COVID-19 occurs in individuals after SARS-CoV-2 infection, usually 3 months from onset of COVID-19 with symptoms that last for at least 2 months that cannot be explained by another clinical entity (8). Several limitations exist in terms of definitions for PASC especially due to the lack of systematic description (6) making it difficult to truly characterize patients presenting this syndrome. Since neurological manifestations are not specifically defined, it is difficult to stratify the study population. Furthermore, a potential confounding factor could be the influence of vaccination on physiological variation of biomarkers in COVID-19 patients, including neuro-PASC patients. To our knowledge, none of the studies have considered the effects of vaccination on the study population. In fact, a few studies specified that recruitment of their study participants was made before the availability of COVID-19 vaccines (41, 46, 68). Therefore, more studies need to be conducted to assess the influence of biomarkers in vaccinated and non-vaccinated population presenting neurological sequalae. Additionally, since GFAP, NfL and tau proteins are presently being used as biomarkers in neurodegenerative diseases, there is also a need to distinguish neuro-PASC from early neurodegenerative processes (69). Furthermore, although there are established relationships between blood and CSF measurements for these markers in other diseases, this has not been thoroughly established for COVID-19 (47).

An important limitation is also the small size of participants in studies (32, 38, 39, 41, 48), which may not accurately reflect the potential future applicability of these biomarkers in a clinical setting. Replication of findings in a larger and more diverse cohorts with distinct phenotypic clusters of symptoms (subgroups) may be a first step toward identifying reliable biomarkers. This could also give some much needed insight into the pathobiology of neuro-PASC, as nervous system affection in COVID-19 and neuro-PASC remains elusive (70). Acute neurological dysfunctions may be caused by direct viral invasion, para-infectious complications, secondary neurological manifestations of systemic disease, or coincident neurological dysfunction in the context of high SARS-CoV-2 prevalence (71). A deeper understanding of the molecular underpinning of the disease will be a linchpin in the discovery of clinically relevant biomarkers. Future large-scale studies should also look to delineate whether SARS-CoV-2 infection affects the levels of biomarkers in the absence of neurologic sequelae (41) to ensure their specificity. Furthermore, a full neurological, psychiatric, and cognitive evaluation as well as neuroimaging data would be ideal; something that was not available or feasible in many studies (32, 36, 38, 48). Studies that include such objective measurements are likely to be more informative and are urgently needed. Ultimately, more research is needed to evaluate the usefulness of these biomarkers in neuro-PASC (72). Moreover, the highlighted biomarkers herein are not the only prospective biomarkers; others have been identified and should be considered in studies looking to identify or validate potential biomarkers (73).

## 4. Conclusion

A handful of studies have explored the measurement of biomarkers NfL, GFAP, tau proteins, IL-6, IL-10, TNF-α, and CPR during acute COVID-19 and PASC. In some cases, higher levels were identified in patients with neurologic symptoms; however, other studies have not corroborated these findings. Ultimately, more research is needed to evaluate the usefulness of these biomarkers in neuro-PASC. Longitudinal clinical, biological, and neuropathological studies are required to better understand the long-term consequences of SARS-CoV-2 infection on the brain and the identification of clinically relevant biomarker in neuro-PASC. Presently, the use of these biomarkers in diagnosing and prognostication neuro-PASC remains tenuous.

## Author contributions

DC and MM drafted the manuscript under the supervision of LC-W. GAR and LC-W contributed to writing and editing the manuscript. All authors contributed to the article and approved the submitted version.

## Funding

This work was supported by grants from: the New Brunswick (NB) Health Research Foundation (NBHRF) and the Centre de Formation Médicale du NB (DUO research grant # DUO2023). GAR is supported by the Canadian Cancer Society (Grant # CHA-22).

## Conflict of interest

The authors declare that the research was conducted in the absence of any commercial or financial relationships that could be construed as a potential conflict of interest.

## Publisher’s note

All claims expressed in this article are solely those of the authors and do not necessarily represent those of their affiliated organizations, or those of the publisher, the editors and the reviewers. Any product that may be evaluated in this article, or claim that may be made by its manufacturer, is not guaranteed or endorsed by the publisher.
